# Addressing sexual and reproductive health and rights with men in prisons: co-production and feasibility testing of a relationship, sexuality and future fatherhood education programme

**DOI:** 10.1108/IJPH-02-2022-0008

**Published:** 2022-07-18

**Authors:** Martin Robinson, Michelle Templeton, Carmel Kelly, David Grant, Katie Buston, Kate Hunt, Maria Lohan

**Affiliations:** School of Psychology at Queen’s University Belfast, Belfast, UK; School of Social Sciences, Education and Social Work at Queen’s University Belfast, Belfast, UK; School of Nursing & Midwifery, Queen’s University Belfast, Belfast, UK; The Senator George J Mitchell Institute for Global Peace, Security and Justice, Queen’s University Belfast, Belfast, UK; MRC/CSO Social and Public Health Sciences Unit, University of Glasgow, Glasgow, UK; Institute of Social Marketing, University of Stirling, Stirling, UK; School of Nursing & Midwifery, Queen’s University Belfast, Belfast, UK

**Keywords:** Relationship and sexuality education, Intervention development, Young offenders, Masculinities, Fatherhood, Gender equality, Co-production, Prison health

## Abstract

**Purpose:**

Young incarcerated male offenders are at risk of poorer sexual health, adolescent parenthood and lack opportunities for formative relationship and sexuality education (RSE) as well as positive male role models. The purpose of this paper is to report the process of co-production and feasibility testing of a novel, gender-transformative RSE programme with young male offenders to encourage positive healthy relationships, gender equality, and future positive fatherhood.

**Design/methodology/approach:**

Using a rights-based participatory approach, the authors co-produced an RSE programme with young offenders and service providers at two UK prison sites using a sequential research design of: needs analysis, co-production and a feasibility pilot. Core components of the programme are grounded in evidence-based RSE, gender-transformative and behaviour change theory.

**Findings:**

A needs analysis highlighted the men’s interest in RSE along with the appeal of film drama and peer-group-based activities. In the co-production stage, scripts were developed with the young men to generate tailored film dramas and associated activities. This co-production led to “If I Were a Dad”, an eight-week programme comprising short films and activities addressing masculinities, relationships, sexual health and future fatherhood. A feasibility pilot of the programme demonstrated acceptability and feasibility of delivery in two prison sites. The programme warrants further implementation and evaluation studies.

**Originality/value:**

The contribution of this paper is the generation of an evidence-based, user-informed, gender-transformative programme designed to promote SRHR of young male offenders to foster positive sexual and reproductive health and well-being in their own lives and that of their partners and (future) children.

## Introduction

The sexual and reproductive health and rights (SRHR) of women and girls are a cornerstone of the United Nations (UN) Sustainable Development Goals, an essential bedrock to people’s health and survival, to gender equality and to economic development (United Nations, 2015). Recognised also is that progress towards women’s and girls’ SRHR requires working with men and boys to challenge unequal gender relations that sustain deficits in SRHR (Kato-Wallace *et al.*, 2016; Starrs *et al.*, 2018; WHO, 2007). However, systematic reviews commissioned by the World Health Organization (WHO) of the evidence suggest that supportive programming with men and boys on SRHR which can challenge gender inequalities known as “gender-transformative interventions” has been slow to develop (Ruane-McAteer *et al.*, 2019, 2020). In addition, to the need to work with men to address global deficits in women’s and girls’ SRHR, the WHO, amongst others, also recognises the need to address health inequalities *among men* and especailly deficits in the SRHR of disadvantaged men (Starrs *et al.*, 2018; WHO, 2018a).

Young men in prisons are especially marginalised, with complex health and social care needs, associated with the intersectionality of socio-economic deprivation, ethnic conflict, ethnic discrimination and adverse childhood traumas, including gender-based violence and intimate partner violence (Lennox, 2014; Naravage *et al.*, 2022; Nascimento *et al.*, 2018; Plugge *et al.*, 2017). Young men in prison are known to have higher rates of sexually transmitted infections and blood-borne viruses relative to non-incarcerated populations (Dolan *et al.*, 2016; Kelly *et al.*, 2020; Rumble *et al.*, 2015) and high rates of adolescent parenthood (Buston *et al.*, 2012).

The UN Convention of the Rights of the Child stipulates that children and young people have the right to high-quality comprehensive relationship and sexuality education (RSE) to build the foundations of positive, safe intimate relationships and healthy lives. International human rights standards under this Convention require that governments guarantee adolescents’ rights to health, life, education and non-discrimination by providing them with comprehensive sex education in primary and secondary schools that is scientifically accurate and objective, and free of prejudice and discrimination (UN Committee on the Rights of the Child [UNCRC], 2016). Furthermore, scientific research demonstrates that young people themselves express the desire and need for comprehensive RSE (Lohan *et al.*, 2018a, 2018b; Pound *et al.*, 2016). However, owing to high levels of school attrition and exclusion among prison populations, many young men in prison lose out on the opportunity of RSE generally afforded to young people (Fields and Toquinto, 2016). Young men in prison also report the lack of involvement with positive male roles in their lives including the role-modelling of positive intimate relationships and involved fatherhood (Buston *et al.*, 2020).

Reported in the scientific literature are evaluations of interventions which do partially address male prison populations’ needs for RSE. Most notably, parenting interventions are increasingly common, and research shows that young fathers in prisons enthusiastically engage with parenting education, and delivery of such courses addresses problems faced by this marginalised group and their families (Armstrong *et al.*, 2017; Buston *et al.*, 2020; McAllister *et al.*, 2012; Meek, 2007). However, existing research shows that parenting programmes are offered to those who are *already* fathers and focus on parenting elements of RSE, rather than as an early intervention with those who are not yet fathers (Buston *et al.*, 2012). The literature also reports evaluations of compulsory programmes, for example “court-mandated batterers programmes” targeted to sex offenders and those perpetrating intimate partner violence (Taylor and Sullivan, 2007). These programmes are not aimed at the general youth offender population. Hence, organisations such as The Howard League have suggested that RSE should be a compulsory component of release and resettlement procedures within prisons (The Howard League, 2016). Yet, this too may be too little too late, overlooking opportunities for education throughout the prison estate and prison sentence.

Building on the systematic review evidence on acceptable and effective RSE (Bailey *et al.*, 2010; Bonell *et al.*, 2013; Guse *et al.*, 2012; Ketting *et al.*, 2015; Kirby, 2002; Pound *et al.*, 2016a; UNESCO and UNAIDS, 2018; United Nations Population Fund, 2015; WHO, 2018b) and our team’s prior research on developing male engagement RSE in schools (Lohan *et al*., 2022, Aventin *et al*., 2020; Templeton *et al*., 2019b; Lohan *et al.*, 2018a, 2018b) and sexual health promotion in prisons (Kelly *et al*., 2020; Templeton *et al*., 2019a), we report the co-production and feasibility testing of a relationship, sexuality and future fatherhood programme for young men (aged 16–21) in young offender centres called *If I Were a Dad.* The aim of the study was to co-produce and conduct a feasibility evaluation of this programme at two national young male offender institutes, one in Northern Ireland and one in Scotland.

## Methodology

### Study design

The research design was informed by a *rights-based approach* (RBA)(United Nations Sustainable Development Group, 2003), a participatory based methodology that shares the broad principles of other participatory research approaches to engaging end users in study design and study outcomes (Bagnoli and Clark, 2010), with three additional requisite principles:

1. The goal must further the realisation of human rights.
2. The process must be guided by human rights standards and principles.
3. The outcome should strengthen the capacity of state agents (duty-bearers) to meet their obligations, and rights-holders to claim their rights, via the processes of empowerment and accountability (United Nations Sustainable Development Group, 2003).

The use of an RBA approach in this study acknowledged that SRHR for these young men is not merely a public health outcome, and is instead related to a broader context of community and institutional rights violations (Mahoney, 2006). Secondly, it was to communicate the human rights basis of enabling men in prison to participate in generating a prison environment that can enhance their SRHR and that of those in the communities to which they return. To implement this RBA, the research design involved three interconnected stages: *A needs analysis with young men and prison environment*, *co-production of the intervention* and *feasibility and acceptability testing.*

### Research setting

The study was conducted in the national young offender institutes of Northern Ireland (Hydebank Wood College) and Scotland (Her Majesty’s Young Offender Institute, Polmont). Site 1 (Hydebank) is home to approximately 120 young men aged 18–21 years, with a separate smaller site for female prisoners. Site 2 (Polmont) has a capacity for 721 prisoners; in 2018–2019, at the time of the study, 325 men were in residence. Young female prisoners are held in a separate establishment. Sentences for young offenders range from six months to life; those serving longer sentences move to adult prisons at age 21. Both sites house people “on remand”, those incarcerated but not yet sentenced. Over 95% of both sites’ populations identify as “white”, including white Irish “travellers”, and while the proportion of Black, Asian, and Minority Ethnic prison populations has been rising, the low proportion reflects the relative ethnic homogeneity recorded in the population census of Scotland and Northern Ireland (National Records of Scotland, 2011; Northern Ireland Statistics and Research Agency, 2011).

### Participants, data collection and analysis

Table 1 summarises the participant recruitment and data collection at each site for the three stages of the research:

#### Stage 1: needs analysis.

Stage 1 was conducted in Hydebank only. It involved four components.

1. *Pilot delivery* of an RSE programme. This was a previously developed male engagement gender transformative RSE programme for use in schools entitled *If I were Jack* (Lohan *et al.*, 2018a, 2018b; Lohan *et al*., 2022). The purpose was to identify the aspects of RSE programming that participants found useful and engaging and the aspects they did not. The pilot delivery of this five-session programme was co-facilitated by a research team member (MT) with a staff member of Barnardo’s, a charity experienced in youth-centred programme delivery in prisons, and our prior selected delivery partner. Recruitment was through prison staff approaching young men and asking for volunteers to participate. Exclusion criteria set by the prisons were prisoners who were sentenced for sex crimes with children, and remand prisoners, who can be released at very short notice. The five-session programme was delivered to 47 participants in small groups of 4–6 men twice a week.
2. *Focus group (FG) interviews* with young men who had participated in the programme and were available at the time of the focus group.
3. *In-depth participant interviews* were conducted with programme participants to delve deeper into their own hopes and desires for current and future intimate relationships, possible parenthood and what part RSE might play in their lives.
4. *Structured interviews* held with prison and Barnardo’s staff, to garner views as continuing collaborators in the design and delivery of the programme.

Written informed consent was obtained by the researchers prior to programme participation and all interviews being conducted. Data were transcribed verbatim, carefully removing any identifiers of participants or their families, and analysed independently and synergistically by two authors (MT and ML) using thematic analysis (Braun and Clarke, 2006).

#### Stage 2: co-production.

The results of the *needs analysis* phase were used to co-produce a new programme with young men. It involved two core components: co-production workshops and programme refinement sessions.

1. *Co-production workshops* involving conceptualisation of programme, development of film scripts and ideas for follow-up activities. Volunteer participants were recruited by prison staff followed by an information meeting with researcher in Site 1 or Barnardo’s staff (Site 2). The co-production sessions involved the research team (MT), a theatre director (DG) and a film producer. DG facilitated participants to create short dramatic scenes illustrating intimate situations and situations of domestic conflict and MT played the role or relevant female characters such as partners or relatives. A Forum Theatre-based approach (Boal, 1992) was then used to allow participants to suggest a range of approaches that might be used to resolve the conflict and the group considered the advantages and disadvantages of each. These scenarios contributed directly to the scripting process both in terms of ideas and the authenticity of the language the counters used. Lifesize paper puppets manipulated by the participants themselves served to represent children within the scenarios, often to quite moving effect. Scripts produced by the director were brought back to the men in both sites involving script readings and suggestion of changes to reflect views and vernacular. The production company also showcased pictures of locations, casting videos of actors, and samples of the draft videos.
  While the production team produced films, the researchers continued to work with the young men on programme activities to accompany the films. Selected activities focussed on areas defined by the young men in the needs analysis stage and views expressed in these co-production workshops about what they would like to do and boundaries on this. Equally, the workshopped ideas were informed by researcher knowledge of the systematic reviews evidence of effective RSE programming components (Bailey *et al.*, 2010; Bonell *et al.*, 2013; Guse *et al.*, 2012; Ketting *et al.*, 2015; Kirby, 2002; Pound *et al.*, 2016; UNESCO and UNAIDS, 2018; United Nations Population Fund, 2015; WHO, 2018b) as well as broader underlying programme behaviour change theories (Ajzen, 1991; Michie *et al.*, 2011) and gender-transformative programming approaches with an emphasis on addressing masculinities and challenging gender inequalities (Haberland, 2015; Ruane-McAteer *et al.*, 2020; WHO, 2011). The confluence of knowledge exchanged led to the selection of activities that afforded opportunities for reflection and communication and skills building, additional culturally-sensitive digital films and resources to generate discussions, and a ‘whole prisons approach’ which would enable facilitation of links with sexual health services and broader alcohol and drug reduction programmes.

1. *Programme refinement* involved implementation and review of the programme prototype by programme delivery team (Barnardo’s) with a further group of men in both sites. Approximately half the participants in both sites had been involved in initial co-production workshops. The research team conducted observations during delivery in both sites (MT and KB), and evaluation focus groups and interviews with participants and implementers. Interview data was transcribed and thematically analysed as above in combination with observational notes. Following this, a detailed manualised programme was developed which included instructions on running the programme and facilitator tips for working with the men on sensitive issues.

#### Stage 3: feasibility pilot.

The aim of this stage was to deliver the new pilot programme as intended in both sites to assess its acceptability and feasibility for future rollout. This stage involved two components.

1. *Delivery of the programme* by Barnardo’s, eight sessions over eight weeks with groups of men not previously involved. Participants were recruited following course handbook instructions with facilitators speaking to volunteers in advance.
2. *Focus group interviews*, and *individual interviews* evaluating the experience of participants and implementers. Permission was obtained from interviewees to take verbatim written notes during discussions.

### Ethical approval

This study was given ethical approval from Office of Research Ethics in Northern Ireland (ORECNI - REC reference: 18/NI/0090, IRAS project ID: 243577).

## Results

### Stage 1: needs analysis

The key findings arising from stage 1 needs analysis related to: young men’s overall impression and motivations in relation to piloted RSE programme (*If I were Jack*) and recommendations on programme content and format.

1. Overall Impression and Motivations

The young men appreciated the opportunity to talk about sex, intimacy and relationships and saw the value in the lessons imparted by the programme.

I learnt new things and thought about things I never had before like what I would do if that happened to me. (Participant, FG 2)

It made you think more about understanding the girl’s point of view and how it [having a baby] affects your life. (Participant, FG1)

The interactive film in this programme worked well for the men, in that it was enjoyable. However, the main protagonist in *If I were Jack*, a 16-year-old youth in school who is shocked and troubled by the news that his girlfriend is pregnant, was regarded as too young and inexperienced for this group of 16–21 year olds to identify with. The strong message from the young men was that they would know just what to do. “I would know what to do like, be there and help out” (Participant, FG4).

Why was he freaking out? It’s not that big a deal, it happens, get on with it. (Interview 8)

Moreover, this group of young men appeared largely disinterested in preventing an “unintended” pregnancy. Instead, they wanted to become fathers and be “good fathers”. They conveyed fatherhood as something to aim towards, something that would *give* them responsibility in their lives, or *make* them responsible and, not least, something that would bring love into their lives.

Being a father means everything, responsibility, being proud of something, happy, be living the glory. (Interview 10)

I’ve never had an experience like that there [birth of a child]. No responsibility like. I’d love to have that there. You know what I mean, a son or daughter, I’d love it so I would. Like picture if you have a daughter about seven or something coming up to you and being ‘daddy I love you’ and all, I mean I would love to hear that. (Interview 3)

Reflecting on what they regarded as their own poor role models of fatherhood, they particularly expressed a desire to be a “better” father, but there were very aware of their own limitations in this regard. They knew they had to work on themselves to be “better men”, by addressing their “temptations”, their drugs and alcohol addictions and their own self-esteem and relationships. They knew this was a struggle.

The way I’m looking at it […] I can’t sort him [a child] out until I sort myself out. (Interview 15)

See I never had a father like, so I want to be a father but I’ll only be a father to a kid and not a mother. I want him to have both. If the girl is taking drugs or committing crimes, I can’t say nothing like I’m in jail; but once I get out of here I’m not, I’m stopping, and if the girl is committing crimes then I’m not going with her. (Interview 13)

#### 1. Recommendations on programme format and content.

The participants identified a need to shorten the film sessions, and remove any *need* to write. Participants signalled these recommendations to reduce fears of young men coming onto the programme with low literacy and reduce any stigma for those with learning difficulties.

Might call you a spastic, put you down or think you’re stupid if you can’t think as quickly as them. You might get up and leave, stops you doing stuff you might want to do. (Interview 1)

In relation to content, the young men conveyed that, to be helpful, the programme would have to include a broader range of more complex issues, pitched at older youths, and be more relevant to their lived experiences and information needs. These included being a man, respect for women, sexual consent sexual health knowledge, shared parenting, together and apart, the impact of drugs alcohol and gambling on relationships. Finally, the formation of the group was very important to young men and informed the next stage. Before joining the *If I were Jack* pilot programme, each of them wanted to know who would be in the group, to be able to avoid conflict and to be able to be relaxed and ‘have a laugh’.

### Stage 1: needs analysis with prison service and delivery partners

Delivery partners and staff in the prison affirmed the value in addressing these issues, providing knowledge/skills training on relationships and sexuality with young men in the prison:

We would be tending to work were those sort of relationships have been part of the offending behaviour as opposed to just general life. (Psychology services)

I’ve noticed the guys taking this programme are a wee bit more open, a wee bit more civil. (Prison officer)

It would be great to see more of this work that is challenging those gender stereotypes across the board because even an awful lot of crime is very gender-based, isn’t it? (Delivery partner)

Recommendations for programming content extended those of the young men, namely, the need for support around the programme in the residential areas of the prison through prison pastoral care support and a suggestion by prison management that prison staff could co-deliver alongside Barnardo’s. The latter suggestion was not taken up immediately recognising imbalances of power between staff and young offenders, though we returned to this suggestion following the pilot study.

### Stage 2: results of co-production

**Figure 1:**
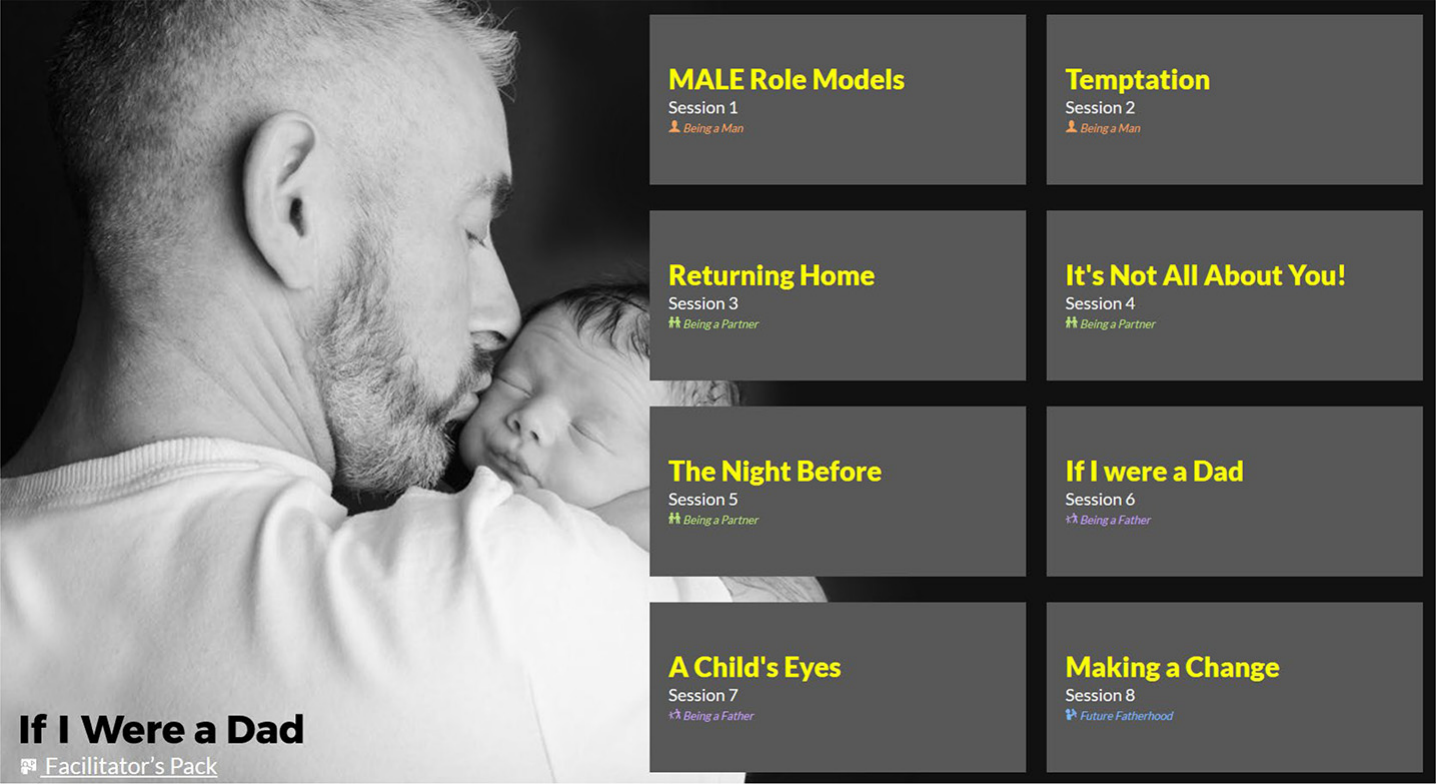


The results of this phase of work was the co-production of a new RSE resource, *If I Were a Dad*, for use with males in young offender units (see Figure 1). Appendix 1 describes the logic model of the programme. Appendix 2 describes the resulting key components of the programme using the “Template for Intervention Description and Replication” (TIDiER) guidelines (Hoffmann *et al.*, 2014). Appendix 3 provides details on the Programme sessions depicted in Figure 1.

### Stage 3: feasibility pilot

Results showed that it was feasible to recruit and schedule participants and for Barnardo’s to deliver the eight-week programme in 2019 in both young offender prison sites. Seven participants began the programme in Site 1 and five completed. Two left on the first day as they felt unsettled in the group; there had been an external dispute in the residential area and one participant had a black eye. Five participants began and completed the programme in Site 2.

(i) Young men’s perceptions of the programme

Overall, the young men at both sites had favourable perceptions of the programme. They saw the programme as novel and commented that it made them think about the future.

I think this was the first thing I done like about being a dad. So, I think it’s obviously opened my eyes a bit. So, I know obviously when I have a wean [child], I know the way I want to be. (Participant, Site 2)

[]I really hate this programme. It wraps all my issues up into one ball and shows me them. I hate it but I love it and need to do it. (Participant, Site 1)

The young men themselves brought up the novelty of discussing these issues in a group, and while bearing in mind the careful prior work by facilitators in composing groups, the group aspect was viewed favourably.

In one-to-one courses it’s more confidential. Like in this group we had a laugh and did get to talk about things we would never before with each other. (Participant, Site 1)

The thing I liked the most was the group work […] Everyone had different thoughts on stuff. (Participant, Site 2)

Young men reported managing their own degrees of disclosure in the group setting and were encouraged to do this during the “ground rules setting exercise”.

You might think ‘oh he’s your friend’ and he is sort of but you’re not going to trust him with too much deep shit, you can only trust yourself in here. (Participant, Site 1)

The young men felt that the drama aspect of the programme was particularly appealing and realistic, and set this programme apart from others.

Aye, aye, I think it was quite realistic – the character and all the situations and that. (Participant, Site 2)

It is obviously based on real life, you know what I mean. Obviously I think is were good for us all to see it. That’s what happens, you know what I mean. … It’s not all happy families. (Participant, Site 2)

However, in both groups there were comments that the female protagonist was not attractive, though realistic.

She [Lisa] pulls the same faces. You know she’s gonna start. (Participant, Site 2)

You have to think about it from her point of view. She has to learn to trust him again and know that she can depend on him. (Participant, Site 1)

While the focus of this stage of the research was on acceptability and feasibility of the programme, not on evaluation of impact on the young men’s lives, within these interviews, young men shared their self-perception of what it meant to them:

[You] feel like you can make something of yourself, it’s all about choices in life. (Participant, Site 2)

If I had of done this course before my daughter was born, I wouldn’t be here [in prison], I would have settled down. (Participant, Site 1)

It’s easy to be a dad but it takes a father to be there. (Participant, Site 2)

This physically shows you how to be a dad and man. Responsibility, jobs and that. You don’t get anything like that out there. (Participant, Site 2)

Yet, participants also pointed to some of the challenges for change, and especially gender-transformative change in relationships with women. This was made obvious when the young men were discussing the female protagonist in the films. One of the young men said:

She’s an aul cunt. Slip her a few slaps to shut her up. (Participant, Site 1)

The group laughed at this comment and the female lead researcher just let the comment settle in order not to disrupt the honesty of feedback. In later discussions with programme facilitators below, they emphasised that while these views were normalised in some men’s lives, their motivation for introducing this and related programmes is the opportunity to challenge harmful masculinities and harmful relationships with men.

(ii) Feedback from Delivery partners and Prison Staff.

The facilitators viewed the programme as feasible to deliver and their impression was that the young men were engaged. Perhaps important to note, there was no incentive for facilitators to be inclined to be favourable. While Barnardo’s is commissioned to do youth work relating to parenting in prisons, there was no additional investment to deliver this programme, rather than an existing programme of their own.

We really enjoyed delivering it. (Facilitator, Site 2)

You put on the videos and there isn’t a peep out of them, they’re watching it, taking it all in and as soon as it’s finished then the discussion gets going. (Facilitator, Site 1)

Equally, the prison management regarded the programme as feasible to deliver with a strong desire to continue to embed the programme as part of their educational opportunities.

Boys turned up every week which speaks volumes. (Prison Management, Site 1)

The results of this partnership should be a win for Hydebank and Polmont.(Prison Management, Site 2)

The key recommended change to enhance feasibility of delivery by the facilitators was that the programme could be optimally delivered by two facilitators, rather than one. There was also a growing acceptance that the second person could be a prison officer, where suitably trained in facilitation skills, returning to a recommendation made by the prison service during the *stage 1: needs analysis.* This was both because programme recruitment was more time-consuming than envisaged (for example, facilitators briefing young men on the programme but also ensuring their scheduling on the programme). Equally, it was because delivery work was regarded as rich and challenging, but at times potentially distressing for the young men and having a second facilitator allowed greater opportunities to address potential distress.

Having two [facilitators] lets you take them out into the other room if it’s getting to them. If you are on your own you couldn’t do that. (Facilitator, Site 2)

There was a growing acceptance of co-operation and learning between youth service providers and prison staff in potential joint delivery with the young men.

It enhances the skillset of the officers […] It shows staff in a new light and softens the delivery from officers and builds relationships with the boys. (Prison Staff, Site 2)

We can identify a staff member to work alongside Barnardo’s to learn and deliver the programme and enhance our philosophy of a whole prisons approach to rehabilitation and education for young men. (Prison Staff, Site 1)

In conclusion, the feasibility pilot of the programme allowed us to complete the final two pieces of TIDiER Intervention design checklist, Modifications and How well it Worked (See Appendix 2).

## Discussion

The contribution of this paper is the generation of an evidence-based, user-informed, gender-transformative programme designed to promote SRHR of young male offenders to foster positive sexual and reproductive health and well-being in their own lives and that of their partners and (future) children. This paper’s contribution is two-fold. Firstly, it illustrates the case that co-production of interventions based on a RBA is important, especially for marginalised groups (Broberg and Sano, 2017; Pyett, 2002). The paper demonstrates how a RBA guided a sequential approach to intervention design involving:

- listening to the needs of those whose rights we sought to fulfil – i.e. young men in prison alongside those of the duty bearers (prison service and delivery partners) who could contribute to these rights;
- the co-production of the intervention logic model and intervention components with young men in prison, and incorporating systematic review evidence on what generates the most effective RSE; and
- a feasibility pilot of the intervention to confirm whether it was acceptable to young men and feasible to deliver in the prison setting.

Though it is widely acknowledged that greater scientific attention to the *development* of interventions, and evidence of co-operation between researchers, end users and practitioners is necessary prior to expensive evaluation studies (Skivington *et al.*, 2021), this scientific process of co-operative design and development is rarely explicated.

The second contribution is a novel RSE programme for young offenders which is ready for further adaptation, implementation and scientific evaluations. Good relationships are understood to be key to prisoner rehabilitation and breaking the cycle of the inter-generational transmission of criminal behaviours (Ladlow and Neale, 2016; Shannon and Abrams, 2018). *If I Were a Dad* is novel in acknowledging the RSE needs of all young men in prison, shifting the focus beyond sexual crime perpetration programmes and parenting programmes towards an early intervention relationships and sexuality programme designed to prepare for future relationships and contemplation of parenthood. The underpinning gender-transformative theory guiding the development of the content of the intervention is aimed at engaging and appealing to young men, while also challenging men to confront harmful practices of masculinity that adversely impact on women, children and other men around them. While the application of this theory was prompted by systematic review evidence that RSE programmes that included a gender and power perspective were more effective than those that did not (Haberland, 2015), as well as broader endorsement of this approach by the WHO (2018), UNESCO (2018) and UNFPA (2014, 2015), it was equally prompted and endorsed by young men’s expressed desires to be “better men” and “better fathers”. The development and use of films scripted from aspects of male prisoners’ own lives and guided activities are underpinned by behaviour change theory (Ajzen, 1991), motivational factors and a broad range of social influences (Michie *et al.*, 2011; Tuong *et al.*, 2014). The culturally attuned films act as “hooks” to engage with men and present key opportunities to reflect upon and build positive sexual and reproductive lives.

### Limitations

The participants in this investigation were restricted to two UK prison sites in Northern Ireland and Scotland. Further research is needed on the acceptability of this intervention in other contexts. Every effort was made to engage as wide a group as possible over the three sequential phases of the research. Nonetheless, as participation in this intervention and its development was entirely voluntary, it is possible that those with greater power or engagement within prison processes are overrepresented in this sample (Steen *et al.*, 2018). Acknowledged is that not all views and needs of those most disadvantaged are necessarily represented, and this may be especially so of men who identify as non-heterosexual.

### Conclusion

The study contributes to gaps in international health policy for the co-production of gender-transformative programming on SRHR with men and boys and especially for marginalised men, such as male prisoners. This study contributes a novel co-produced RSE programme created *with* male young offenders *for* male young offenders to promote the SRHR of male prisoners, their partners and gender-equality. It used a rights-based study design, demonstrating high acceptability and feasibility for delivery in two young offender sites. Further work is now needed to examine the extent to which this intervention may be successful in effecting behaviour and attitudinal change to promote SRHR for young male offenders and the communities to which they return over the short to medium term.

### Highlights:

- Using a rights-based participatory approach, we co-produced a gender transformative, film-based interactive programme for young male offenders to promote the sexual and reproductive health and rights of male prisoners, their partners and gender-equality.
- The programme is unique in the scientific literature on prison-based interventions as a gender-transformative relationship and sexuality education programme.
- The programme proved acceptable and feasible to deliver in two national young offender prisons and warrants further implementation and evaluation studies.

## Acknowledgments

The research team would like to acknowledge the help of the young men and staff at Hydebank Wood College and Her Majesty’s Young Offender Institute, Polmont, in the design and development of the programme as well as the contribution of Barnardo’s staff in delivering and providing feedback on the programme. Morrow Communications Belfast produced the film materials used as part of this programme. The authors would also like to thank members of the Stakeholder Advisory Group from the following organisations: Community Justice Scotland, Public Health Agency Northern Ireland (NI), Departments of Health in NI and Scotland, Chief Medical Officer NI, Scottish Government; Fatherhood Institute UK; Families Outside; Probation Board of NI; and Mr Mark Carson –- Lay Advisor.

## Funding

This work was supported by the Medical Research Council (Grant No. MR/RO14450/1).

## Contribution of authors

ML was the Chief Investigator and oversaw all aspects of the study. ML, MT, KH KB and CK designed the study. MT, ML and KB conducted the needs analysis. MT, DG, ML, KH and KB and MR conducted the co-production stage. ML, MR, KB and KH undertook feasibility analysis. MR, ML and CK drafted the manuscript and all co-authors contributed to writing and editing of the manuscript and approved the final version.

## Figures and Tables

**Figure A1:**
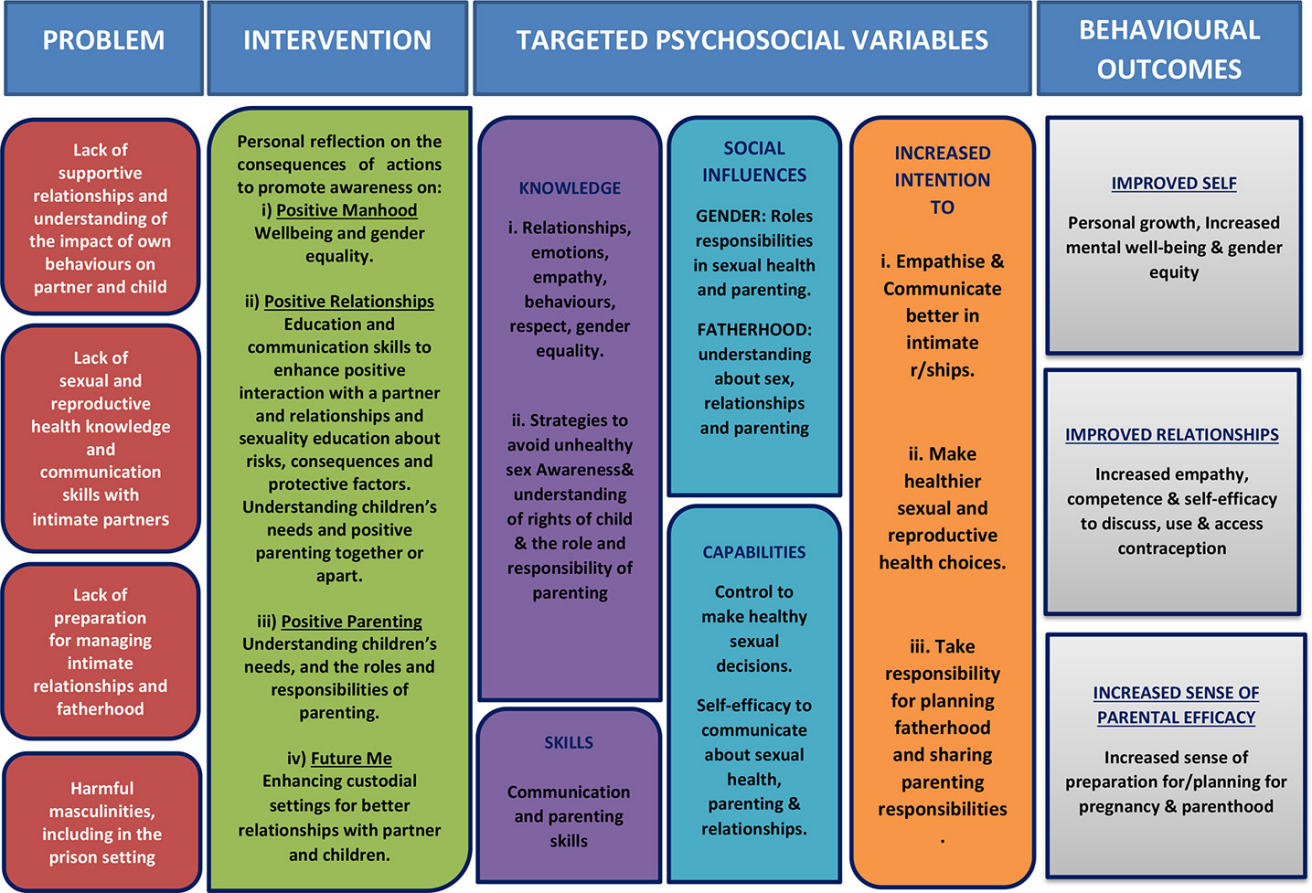
Project Logic Model for If I Were a Dad Programme

**Table 1:** Data sources at each stage of study

Stage	Site	Participants (young men)	Participants (prison staff and Barnardo’s delivery partners)
*1. Needs analysis*	Hydebank (Site 1)	15 pilot sessions of *If I Were Jack* (*n* = 47) 20 in-depth interviews (*n* = 20) 8 focus groups (n = 25)	Interviews (*n* = 8)
*2. Co-production*	Hydebank	8 pre-design sessions (*n* = 4 participants) 8 refinement sessions (*n* = 4 participants) 1 session observation 1 focus group interview (*n* = 3 participants)	Interviews (*n* = 2)
	Polmont (Site 2)	1 pre-design session (*n* = 8) 8 refinement sessions (*n* = 8 participants) 2 session observations 1 interview	1 interview 1 paired interview
*3. Feasibility pilot*	Hydebank	Full delivery of programme (8 sessions with 5 participants) 1 focus group at outset (*n* = 7) 1 focus group at end (*n* = 5)	Interviews (*n* = 2)
	Polmont	Full delivery of programme by delivery partner; 8 sessions with 7 participants) 1 focus group at end (*n* = 4)	3 interviews

**Table A1:** Programme themes and content overview

*Session*	*Theme*	*Aim & Intended Outcome*
1. Male Role Models	Being a Man	To examine and challenge gender stereotypes faced by young men. The intended outcome is improved knowledge and attitudes about gender-based issues
2. Temptation	Being a Man	To examine pressures faced by young men and how these can affect one’s self and others. The intended outcome is greater resilience and skills to make more positive decisions regarding behaviour
3. Returning Home	Being a Partner	To explore the topic of conflict in relationships and how this may be handled. The intended outcome is to increase skills and ability to address relationship conflict
4. It’s Not All About You!	Being a Partner	To raise awareness of issues around sexual consent and human rights. The intended outcome is better preparedness for healthy sexual relationships and future fatherhood
5. The Night Before	Being a Partner	To examine issues around sexual and reproductive health. The intended outcome is greater understanding of positive and healthy sex and of how to potentially engage with sexual health services
6. If I Were a Dad	Being a Father	To explore issues around parenting and partnership. The intended outcome is greater knowledge of potential issues around parenting with or without a partner and how one might deal with these
7. A Child’s Eyes	Being a Father	To explore the potential impact of violence on a child and partner. The intended outcome is increase awareness of the impact of violence, and skills to nurture and support a child
8. Making a Change	Future Fatherhood	To encourage participants to share what they have learned and change the prion environment. The intended outcome is a sense of empowerment of participants and promotion of positive attitudes toward masculinity and parenthood in the prison setting

## Appendix 1

Figure A1

## Appendix 2. Intervention characteristics of “If I Were a Dad”

### Brief name

*If I Were a Dad* is an evidence-informed relationship and sexuality education programme, created with and for young men aged 16 –21 years in custodial settings.

### Why

It is intended to improve knowledge, attitudes and skills related to positive masculinities, sexual health, relationships and future parenthood.

The programme is informed by three underpinning theories:

1. *Human Rights Based Approach* (United Nations Sustainable Development Group, 2003) which informed the participatory approach and involvement of the target group as duty bearers in generating conditions supporting human rights to positive health and education.
2. *Gender-Transformative Theory* (WHO, 2011) to both engage with and challenge men to consider constructs of masculinities and the impact of their behaviours on women and children.
3. *Theory of Planned Behaviour* (Ajzen, 1991), informing experiential based “stop-and-think” strategies to build intentions for positive relationships. (See Appendix 1 for programme logic model).

### What

*Materials*: The programme is delivered via an application-based collection of short video dramas scripted from the stories of young men in custody accompanied by a number of classroom activities and materials. The programme content covers four central themes: *Being a Man*, *Being a Partner*, *Being a Father* and *Future Fatherhood* (see Table 1 *and* Appendix 2 *Session and Content Overview*). All materials made available to facilitators electronically online or via USB flash drives.

Films are intended to be watched as a group, to end in “cliff-hanger scenarios” with potentially different ensuing actions, to allow the young men to imagine how they would deal with the situation (Preview the *If I Were a Dad* materials: https://tinyurl.com/IIWADShowcase). This is followed by individual and group activities to closely examine aspects encountered. These discussions and activities are supplemented by a set of classroom resources for facilitators consisting of posters, flashcards, puppetry, and additional videos made with men who are in/have left prison. Links with the sexual health, care and drug and rehabilitation programmes and pastoral care available in the prison setting are signposted. See Appendix 3 for overview of content

*Procedures*: Facilitators are provided a comprehensive manual detailing the programme activities and the purpose or rationale behind these. The manual details recommended adaptation and alternative activities to enable tailoring where necessary.

Enabling and supporting activities for delivery include prison management buy-in, or “whole prisons approach” to:

1. facilitate opportunities to inform men about the programme and facilitate timetabling of the programme;
2. have referral services and debriefing sessions on stand-by, such as contacts for drug/alcohol addiction programmes or pastoral support services and psychological services; and
3. recognise their participation, by a senior prison staff member presenting certificates at the end of the course.

### Who provided

The programme is designed to be delivered by trained facilitators, experienced in delivering youth-centred personal development courses to young men within custodial settings.

The programme manual contains detailed delivery instructions, and a video of three facilitators discussing challenges in delivery.

### How

Sessions are delivered to a small group of young men (n=4-8) by one or two facilitators. Ground rules for the group are formulated collaboratively at the outset, based on programme resources.

### Where

The intervention is designed to be delivered in a room suitable for group work with a computer and large screen/projector to show videos. It may be delivered in or outside of custodial settings. Ideally, there should be a break-out/fresh air area for break times.

### When and how much

The programme comprises eight sessions, lasting approximately 120 min each, with three associated activities divided by comfort breaks.

### Tailoring

The film drama materials presented incorporated imagery confirmed to be acceptable and relevant to the young men during the co-production stage. Some of the programme activities and resources are presented in a Scottish and Northern Irish accent. See for example: Northern Ireland version (https://tinyurl.com/ue4wcsaw) and Scottish version (https://tinyurl.com/5vsd4cc5). The programme was tailored to cater for the transient prison context – with sessions may be viewed independently, yet flow as a journey for those who complete.

### Modifications

It was decided that programme delivery could be optimised through delivery by two facilitators. The facilitation team could include prison officers co-delivering with a youth work team where prison officers could demonstrate non-judgemental, youth-centred facilitation skills.

### How well

The programme was delivered as intended in two sites for the intended duration with groups of men to assess programme feasibility and delivery mechanisms. While there was some drop-off in participation, this was regarded as part of the voluntary nature of the programme and the overall engagement and satisfaction expressed by all involved was high, especially given the context. Future evaluation of impact is warranted.

## Appendix 3

Table A1
